# Hyperreflective Intraretinal Spots in Diabetics without and with Nonproliferative Diabetic Retinopathy: An *In Vivo* Study Using Spectral Domain OCT

**DOI:** 10.1155/2013/491835

**Published:** 2013-12-09

**Authors:** Stela Vujosevic, Silvia Bini, Giulia Midena, Marianna Berton, Elisabetta Pilotto, Edoardo Midena

**Affiliations:** ^1^Department of Ophthalmology, University of Padova, Via Giustiniani 2, 35128 Padova, Italy; ^2^University Campus Biomedico, Via Alvaro del Portillo 21, 00128 Roma, Italy; ^3^Fondazione G.B. Bietti, Via Livenza 3, 00198 Roma, Italy

## Abstract

*Purpose*. To evaluate the presence of hyperreflective spots (HRS) in diabetic patients without clinically detectable retinopathy (no DR) or with nonproliferative mild to moderate retinopathy (DR) without macular edema, and compare the results to controls. *Methods*. 36 subjects were enrolled: 12 with no DR, 12 with DR, and 12 normal subjects who served as controls. All studied subjects underwent full ophthalmologic examination and spectral domain optical coherence tomography (SD-OCT). SD-OCT images were analyzed to measure and localize HRS. Each image was analyzed by two independent, masked examiners. *Results*. The number of HRS was significantly higher in both diabetics without and with retinopathy versus controls (*P* < 0.05) and in diabetics with retinopathy versus diabetics without retinopathy (*P* < 0.05). The HRS were mainly located in the inner retina layers (inner limiting membrane, ganglion cell layer, and inner nuclear layer). The intraobserver and interobserver agreement was almost perfect (*κ* > 0.9). *Conclusions*. SD-OCT hyperreflective spots are present in diabetic eyes even when clinical retinopathy is undetectable. Their number increases with progressing retinopathy. Initially, HRS are mainly located in the inner retina, where the resident microglia is present. With progressing retinopathy, HRS reach the outer retinal layer. HRS may represent a surrogate of microglial activation in diabetic retina.

## 1. Introduction

An increasing body of evidence suggests that retinal neurodegeneration and inflammation occur in human diabetes even before the development of clinical signs of diabetic retinopathy (DR) [1]. Retinal neural cell loss (neurodegeneration) has already been demonstrated *in vivo* (as thinning of retinal nerve fiber and ganglion cell layers), both in type 1 and 2 diabetes [2–7].

Retinal microglia activation has been recognized as the main responsible for the initial inflammatory response, even though the exact mechanism through which inflammatory cytokines are released remains poorly known [8]. Some experimental studies have shown that retinal inflammation occurring during the course of diabetes mellitus is a relatively early event and that it precedes both vascular dysfunction and neuronal degeneration [1, 8]. Joussen at al. demonstrated in animal models of diabetes mellitus that ICAM-1- and CD18-mediated leukocyte adhesion is increased in the retinal vasculature and accounts for many of the signature lesions of DR [1]. Ibrahim et al. demonstrated in rats that the accumulation of Amadori-glycated albumin (AGA) within the 8-week diabetic retina elicits microglial activation and secretion of Tumor necrosis factor alpha (TNF-*α*) [8].

Retinal macroglia and retinal microglia activation have been documented histopathologically and hypothesized *in vivo* [7, 9–11]. The activated microglia secretes cytokines and other proinflammatory molecules used for the phagocytosis and the destruction of damaged cells as well as for the triggering of reparative processes which lead to the formation of glial scars [8]. If microglia remains in an activated state, continuously released cytokines may damage the neighbouring cells particularly the neuronal and the vascular ones, leading to the onset of different retinal changes [8]. According to this hypothesis, some histopathological studies (performed both in animals and in humans) have confirmed the activation of microglial cells, as well as the presence of different inflammatory molecules secreted by microglia, commonly associated with neuronal and endothelial death [9, 10, 12–14].

Spectral domain optical coherence tomography (SD-OCT) has become a valuable tool for the *in vivo* evaluation of single retinal layers (both the inner retina and the outer retina) in diabetic patients [7, 15, 16]. Moreover, it has been used for the evaluation of hyperreflective retinal spots in age related macular degeneration, diabetic macular edema, and retinal vein occlusion [16–21].

The main purpose of this study was to determine, *in vivo*, by SD-OCT, the presence and location of hyperreflective spots in the retina in diabetic patients without DR or with early stages of DR (mild and moderate nonproliferative DR) without macular edema versus normal subjects.

## 2. Material and Methods

36 subjects were included in the study: 12 subjects served as controls, 12 patients were affected by diabetes without diabetic retinopathy (no DR), and 12 patients were affected by diabetes and diabetic retinopathy (mild to moderate). One eye of each subject was used for the SD-OCT analysis. The exclusion criteria were proliferative DR, macular edema, any type of previous retinal treatment (macular laser photocoagulation, vitrectomy, intravitreal steroids, and/or antiangiogenic drugs), any intraocular surgery, refractive error >6D, previous diagnosis of glaucoma, ocular hypertension, uveitis or other retinal diseases, and significant media opacities that precluded fundus imaging. All patients underwent SD-OCT using Spectralis (Heidelberg Engineering, Heidelberg, Germany). A single 180° SD-OCT line scan (6 mm length) centered onto the fovea was analyzed for each patient, looking for the presence of hyperreflective spots. Two red vertical lines were traced at 500 *μ*m and 1500 *μ*m from the center of the fovea in the temporal region, thus excluding the foveal avascular zone. A manual count of the hyperreflective spots, defined as small, punctiform, white lesions, was performed between the two markers. The layering was obtained using the automatic layering of the Spectralis SD-OCT with manual refinement for the boundaries of the most critical layers (e.g., inferior boundary of ganglion cell layer where contrast is lower).

**Figure 1:**
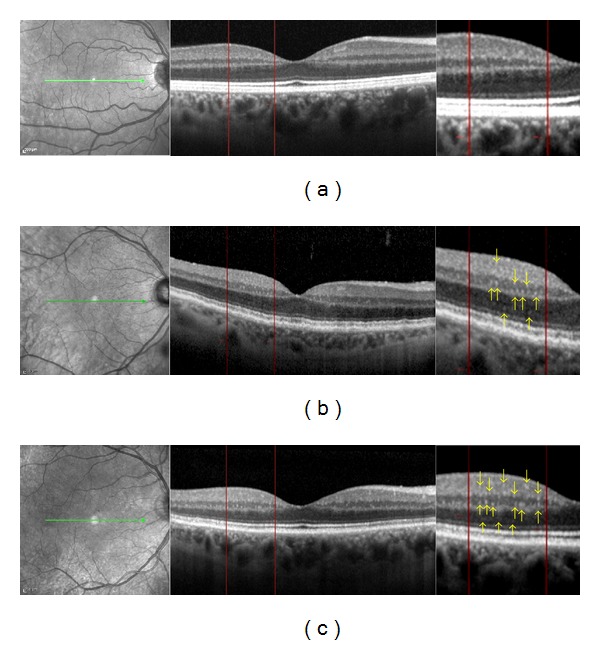
Spectral domain OCT linear scans in the macula of (a) normal subject, (b) diabetic patient without retinopathy, and (c) diabetic patient with mild nonproliferative diabetic retinopathy. Two vertical lines were traced at 500 *μ*m and 1500 *μ*m from the center of the fovea in the temporal region where the count of the hyperreflective spots was performed. The count was performed in the following retinal layers: inner limiting membrane (ILM) to ganglion cell layer (GCL), inner nuclear layer (INL) to outer plexiform layer (OPL), and outer nuclear layer (ONL). The yellow arrows indicate the HRS seen on the magnification image. The number of HRS is higher in diabetic eyes with DR versus diabetic eyes without retinopathy.

The count was performed starting from the inner limiting membrane (ILM) to the outer nuclear layer (ONL), including ILM to ganglion cell layer (GCL); inner nuclear layer (INL) to outer plexiform layer (OPL), and ONL. All measurements were performed by two independent, masked graders (Figure 1).

A written consent form was obtained from all patients as well as the approval from our institutional ethics committee. The study was conducted in accordance with the tenets of the Declaration of Helsinki.

The difference in the number of hyperreflective spots was compared among groups by means of analysis of variance (ANOVA).

## 3. Results

The mean age of the different groups was 55.2 ± 10 years in normal subjects, 56.9 ± 13 years in diabetics without DR (no DR), and 59.3 ± 11.2 years in nonproliferative DR (NPDR). There was no statistically significant difference in the age among the three groups. All diabetics had type 2 diabetes mellitus (DM). Mean HbA1c was 7.6 ± 1.5% in no-DR and 7.8 ± 0.8 in NPDR. Mean diabetes duration was 10.2 ± 4 years.

In the ILM-GCL layer, the hyperreflective spots were present in 3% of control eyes, 55% of no-DR eyes, and 90% of DR eyes. In the INL-OPL layer, the hyperreflective spots were present in 1% of control eyes, 35% of no-DR eyes, and 75% of DR eyes.

In the ONL layer, the hyperreflective spots were present in 0% of control eyes, 10% of no-DR eyes, and 25% of DR eyes (Table 1).

As the number of hyperreflective spots is concerned with the ILM-GCL layer, the mean number was 0.05 ± 0.22 in control eyes, 2.92 ± 1.88 in no-DR eyes, and 6.63 ± 2.5 in DR eyes; in the INL-OPL layer, the mean number was 0.03 ± 0.48 in control eyes, 2.93 ± 1.73 in no-DR eyes, and 8.03 ± 3.18 in DR eyes; in the ONL layer the mean number was 0.0 in control eyes, 1.58 ± 1.31 in no-DR eyes, and 6.18 ± 2.89 in DR eyes (Table 2).

The number of hyperreflective spots was significantly higher in both diabetics with no DR and diabetics with DR versus controls (ANOVA, *P* < 0.05) and in diabetics with DR versus diabetics without retinopathy (ANOVA, *P* < 0.05). The intraobserver and interobserver agreement was almost perfect (*κ* > 0.9) for all measurements.

## 4. Discussion

In this study, we report the presence of hyperreflective spots (HRS), documented by SD-OCT, in the more inner retinal layers (ILM, GCL), in the INL to OPL, and in the ONL in diabetic patients with and without DR. When compared to healthy subjects, these hyperreflective spots were significantly much more numerous in the inner retina of diabetics and completely absent in the outer retina of controls. The HRS have been recently described by some authors, who hypothesized different pathogenetic origin, and who also used two different terms to name these lesions. They named HRS as hyperreflective foci or hyperreflective dots [16–21]. We suggest that the term spots better encompasses the aspect of these lesions, but we do not consider different terms a limitation. Coscas et al. were the first to report the presence of HRS, as small in size, punctiform hyperreflective elements, scattered throughout all retina layers but mainly located in the outer retina layers around fluid accumulation in the intraretinal cystoid spaces in age related macular degeneration, suggesting that they may represent activated microglia cells [18, 19]. Bolz et al. described the HRS distributed throughout all retinal layers (in some cases confluent at the border of the ONL and within the outer plexiform layer) in eyes with different types (diffuse, cystoid) of diabetic macular edema (DME) [17]. Bolz et al. hypothesized that HRS may represent subclinical features of lipoprotein extravasation that act as precursors of hard exudates, as they were not observed on clinical examination, fundus photography, or fluorescein angiography, due to their small size (approximately 30 microns) [17]. Uji et al. reported the presence of HRS in the outer retina (53.7%) and in the inner retina (99.1%) in eyes with DME [20]. The HRS in the outer retina were closely associated with disrupted external limiting membrane and IS/OS line and decreased visual acuity, suggesting an origin from degenerated photoreceptors or macrophages engulfing them [20]. Ogino et al. reported the presence and distribution of HRS in retinal vein occlusion [21]. The HRS were present in all retinal layers (both inner and outer retinas). In most of the eyes affected by serous retinal detachment, the HRS were attached to the external limiting membrane [21]. The authors did not find any sign of hard exudates. They also suggested that the HRS present in affected areas may explain the leakage of blood constituents, whereas the HRS around the OPL in the unaffected areas in retinal vein occlusion were associated with the absorption of water and solutes [21]. Framme et al. reported the presence of HRS in patients with both focal DME and diffuse DME [16]. After anti-VEGF treatment, the HRS were the first features to disappear or to reduce significantly. Therefore, it has been hypothesized that HRS represent a clinical marker of inflammatory response [16, 19].

In our study, the presence and number of HRS increased in diabetics versus normals, and especially in diabetics with clinical signs of DR. HRS were mainly located, at an early stage, into the inner retinal layers, from ILM to GCL layer. This depends on the fact that the resting retinal microglia is physiologically located in the inner retinal layers, and before migrating toward outer layers, the activation process begins into and expands from the inner retinal layers. When activated, microglial cells undergo significant changes as the shape and size of individual cells are concerned and aggregate among them to form the microglial aggregates [10]. Neither of the patients included in this study had macular edema, hard exudates, or subclinical (OCT) signs of DME. Because all our patients were affected by initial stages of diabetic retinopathy (with normal macula) or even diabetic without retinopathy, we are confident in excluding the hypothesis that in our population, HRS represent lipid exudates or degenerated photoreceptors. HRS in our population are more reasonably secondary to early microglia activation (aggregates of microglial cells), already described in experimental studies in diabetic retina [9, 10, 22]. To confirm our hypothesis, a recent study has documented a significant increase in the thickness of IPL and INL versus a decrease in GCL and RNFL, in patients with nonproliferative DR, as signs of macroglial cell activation [7].

Previous studies also showed that low-level chronic inflammation contributes to retinal dysfunction changes during the course of DM [23–26]. Pathologic levels of neuroretinal inflammatory mediators may contribute to the evolution of diabetic retinopathy, up to the proliferative stage [27]. Moreover, Zeng et al, investigating the microglial activation around retinal capillaries in human eyes, detected signs of the so-called “microglial perivasculitis,” a phenomenon secondary to microglial activation. The same group evidenced that this inflammatory local process may secondary affect ganglion cells, suggesting precocious inflammation changes at the level of retinal neuronal cells [10]. These data may be supported by our findings of HRS, which are initially present in most internal retinal layers. The release of inflammatory mediators (including VEGF) provokes the extension of the inflammatory process through the entire retina, increasing both vascular permeability and neuronal damage, thus creating a vicious circle [10]. The spread of activated microglia cells beyond outer retinal layers, through an experimentally documented intracellular mechanism, is also documented by the appearance of HRS up to the level of retinal pigment epithelium [22].

In conclusion, diabetic retinopathy is characterized by retinal microglia activation, previously shown in experimental studies on animals and autoptic eyes. SD-OCT documents discrete reflectivity changes (hyperreflective spots) which may correspond to aggregates of microglia activated cells, as reported in other retinal diseases. The number of SD-OCT hyperreflective spots increases with the clinical progression of DR and shows an inner to outer retina migration. A prospective study on the evolution of DR and HRS location and presence is currently underway in order to strengthen our hypothesis.

## Figures and Tables

**Table 1:** The percentage of eyes with hyperreflective spots.

Retinal microglial activation	Control	Diabetic	
		No DR	NPDR
ILM-GCL	3%	55%	90%
INL-OPL	1%	35%	75%
ONL	—	10%	25%

No DR: diabetic patients without retinopathy; NPDR: nonproliferative diabetic retinopathy; ILM-GCL: inner limiting membrane-ganglion cell layer; INL-OPL: inner nuclear layer-outer plexiform layer; ONL: outer nuclear layer.

**Table 2:** The number of hyperreflective spots.

Hyperreflective spots/number (±SD)	Control	Diabetic	
		No DR	NPDR
ILM-GCL	00.5 (0.22)	2.92 (1.88)	6.63 (2.5)
INL-OPL	0.03 (0.48)	2.93 (1.73)	8.03 (3.18)
ONL	0.00 (0.00)	1.58 (1.31)	6.18 (2.89)

SD: standard deviation; No DR: diabetic patients without retinopathy; NPDR: nonproliferative diabetic retinopathy; ILM-GCL: inner limiting membrane-ganglion cell layer; INL-OPL: inner nuclear layer-outer plexiform layer; ONL: outer nuclear layer.
